# Prothrombin complex concentrate (PCC) for treatment of trauma-induced coagulopathy: systematic review and meta-analyses

**DOI:** 10.1186/s13054-023-04688-z

**Published:** 2023-11-02

**Authors:** Ioannis Hannadjas, Arthur James, Ross Davenport, Charlotte Lindsay, Karim Brohi, Elaine Cole

**Affiliations:** 1 Centre for Trauma Sciences, Blizard Institute, Queen Mary University of London, London, England; 2 GRC 29, AP-HP, DMU DREAM, Department of Anaesthesiology and Critical Care, Pitié-Salpêtrière Hospital, Sorbonne University, 47-83 Boulevard de l’Hôpital, 75013 Paris, France

**Keywords:** Major trauma, Trauma-induced coagulopathy, Prothrombin complex concentrate, Blood coagulation factors, Blood transfusion, Trauma hemorrhage

## Abstract

**Background:**

Trauma-induced coagulopathy (TIC) is common in trauma patients with major hemorrhage. Prothrombin complex concentrate (PCC) is used as a potential treatment for the correction of TIC, but the efficacy, timing, and evidence to support its use in injured patients with hemorrhage are unclear.

**Methods:**

A systematic search of published studies was performed on MEDLINE and EMBASE databases using standardized search equations. Ongoing studies were identified using clinicaltrials.gov. Studies investigating the use of PCC to treat TIC (on its own or in combination with other treatments) in adult major trauma patients were included. Studies involving pediatric patients, studies of only traumatic brain injury (TBI), and studies involving only anticoagulated patients were excluded. Primary outcomes were in-hospital mortality and venous thromboembolism (VTE). Pooled effects of PCC use were reported using random-effects model meta-analyses. Risk of bias was assessed for each study, and we used the Grading of Recommendations Assessment, Development, and Evaluation to assess the quality of evidence.

**Results:**

After removing duplicates, 1745 reports were screened and nine observational studies and one randomized controlled trial (RCT) were included, with a total of 1150 patients receiving PCC. Most studies used 4-factor-PCC with a dose of 20–30U/Kg. Among observational studies, co-interventions included whole blood (*n* = 1), fibrinogen concentrate (*n* = 2), or fresh frozen plasma (*n* = 4). Outcomes were inconsistently reported across studies with wide variation in both measurements and time points. The eight observational studies included reported mortality with a pooled odds ratio of 0.97 [95% CI 0.56–1.69], and five reported deep venous thrombosis (DVT) with a pooled OR of 0.83 [95% CI 0.44–1.57]. When pooling the observational studies and the RCT, the OR for mortality and DVT was 0.94 [95% CI 0.60–1.45] and 1.00 [95% CI 0.64–1.55] respectively.

**Conclusions:**

Among published studies of TIC, PCCs did not significantly reduce mortality, nor did they increase the risk of VTE. However, the potential thrombotic risk remains a concern that should be addressed in future studies. Several RCTs are currently ongoing to further explore the efficacy and safety of PCC.

**Supplementary Information:**

The online version contains supplementary material available at 10.1186/s13054-023-04688-z.

## Introduction

Enhanced trauma resuscitation with major hemorrhage protocols, balanced transfusion, and empiric use of tranexamic acid have improved early survival for injured patients with hemorrhage [1]. However, mortality rates associated with bleeding remain high [2], particularly in cases of uncontrolled hemorrhage and trauma-induced coagulopathy (TIC) [3, 4]. Patients who develop TIC require more blood transfusions, have a higher incidence of multiple organ dysfunction, and have an increased risk of death [5, 6]. Currently, standard component therapy for TIC involves administering tranexamic acid [7], fresh frozen plasma (FFP), and supplemental fibrinogen and calcium [8]. Prothrombin complex concentrates (PCCs) have been proposed for the management of major bleeding and coagulopathy after trauma, particularity when used in conjunction with fibrinogen concentrate to treat low fibrinogen levels [9–11].

PCCs contain vitamin K-dependent clotting factors (II, VII, IX, and X) and are traditionally used for emergency reversal of vitamin K antagonists in major hemorrhage [12]. The products are either 3- or 4-factor-PCC (3F, 4F-PCC) formulations depending on the concentrations of Factor VII [13]. Compared to FFP, PCC has a long shelf-life at room temperature and therefore can be available rapidly for treating clinicians both in-hospital and prehospital settings. It contains a high, supraphysiological concentration of clotting factors and is administered in small volumes. However, the effectiveness of PCCs as a treatment for TIC remains uncertain, and there is a lack of international consensus regarding the indications, timing of administration, adjunct therapies, and dosing protocols. Moreover, it is unclear as to whether PCC’s are a safe alternative to FFP in the early or later phases of major hemorrhage management, with an increased thrombosis risk in patients who are themselves in a pro-coagulant phase post-injury [14–16].

The overall aim of this systematic review was to investigate the outcomes and safety of PCC in major trauma patients with TIC. The primary objective was to characterize the use of PCC administration during trauma hemorrhage. Secondly, we wished to investigate clinical outcomes, specifically mortality and venous thromboembolism (VTE), associated with PCC administration during trauma hemorrhage.

## Methods

### Study design

This systematic review follows the Preferred Reporting Items for Systematic Reviews and Meta-analyses (PRISMA) [17] (Additional file 1**)**.

### Data sources and search strategies

We conducted a systematic literature search to identify publications that examined the use of PCCs in the treatment of TIC in adult trauma patients. The search was performed in MEDLINE (via PubMed) and EMBASE databases using a combination of free text and structured vocabulary (MeSH terms). We also searched ClinicalTrials.gov to identify relevant ongoing or completed randomized clinical trials related to this topic. Both searches included publications released between January 1, 2010, and April 22, 2023. The complete search strategies for MEDLINE, EMBASE, and ClinicalTrials.gov can be found in Additional file 1.

### Selection process

After removing duplicates, two reviewers (IH, AJ) independently assessed the eligibility of retrieved references. Any discrepancies were resolved by discussion with a third reviewer (EC) to reach consensus. Eligibility criteria were interventional and observational studies of adult patients being administered PCC to treat trauma-induced coagulopathy (Full PICO in Additional file 1). We excluded studies dedicated to pediatrics as well as those focusing on PCC administered to reverse anticoagulation, for traumatic brain injury only, or for liver disease. We also excluded case series, case reports, conference abstracts, and studies not published in English. The software Rayyan (rayyan.ai) was used for the title and abstract screening [18].

### Data extraction

Data extraction was performed independently by two reviewers (IH, AJ), and the discrepancies were discussed with a third reviewer (EC) to reach consensus. When adjusted and non-adjusted results were available, only adjusted results were extracted. From each included study, the following data were extracted:

- Information about the study: Main author, year of publication, study title, study design (observational/randomized controlled trial), number of centers (single center or multi-center), number of patients included, country of the first authors, outcomes reported
- Information about the patients: injury type (penetrating or blunt or both, if both then proportion of penetrating injuries), INR at admission, proportion of patients receiving an anti-platelet treatment, injury severity score (ISS)
- Information about the PCC: indication (triggering criteria if reported), timing of PCC administration (in minutes after the trauma), molecule used, dose (in UI/Kg)
- Information about the comparators: molecule used, dose (in UI/Kg)
- Information about co-treatment (co-treatment was defined as pharmaceutical interventions protocolized in at least one group of the study): molecule used, dose (in UI/Kg)
- Outcomes: For each included study, all reported outcomes were retrieved one by one. The primary outcome of this systematic review was in-hospital mortality. We also extracted transfusion volume (red blood cell [RBC] units, platelets units, and FFP units and the proportion of patients with deep venous thrombosis (DVT)).

From each clinical trial protocol data were extracted on the inclusion criteria, the intervention planned and comparator, the main outcome, and current stage reported on clinicaltrials.gov.

### Data synthesis and statistical analysis

Data were reported as either mean with standard deviation or median with interquartile range, as provided by the included studies. We used odds ratio (OR) and 95% confidence interval as a summary measure. Assuming an important heterogeneity among included studies results, a random-effects model was applied, and the Paule-Mandel procedure was used to calculate the heterogeneity variance (*τ*2). Subgroup meta-analyses were conducted for propensity-matched studies and for those without adjustment to explore the impact of the method used on the results published. We considered a *p*-value < 0.05 as significant. All analyses were performed using the R v4.2.1 software.

### Study quality assessment

We used the Risk Of Bias In Non-randomized Studies of Interventions (ROBINS-I) [19] and the Revised Tool for Risk of Bias in Randomized Trials (RoB2) [20] to assess the quality of included observational studies and the randomized controlled trial, respectively. Three of the authors performed the quality assessments independently (IH, AJ, CL), and discrepancies were discussed with a last reviewer (EC) to reach consensus. For each intervention evaluated in the meta-analysis, we rated the quality of evidence for PCC administration according to the Grading of Recommendation, Assessment, Development, and Evaluation (GRADE) Working Group system [21]*.*

## Results

### Selection of the relevant studies

**Fig. 1:**
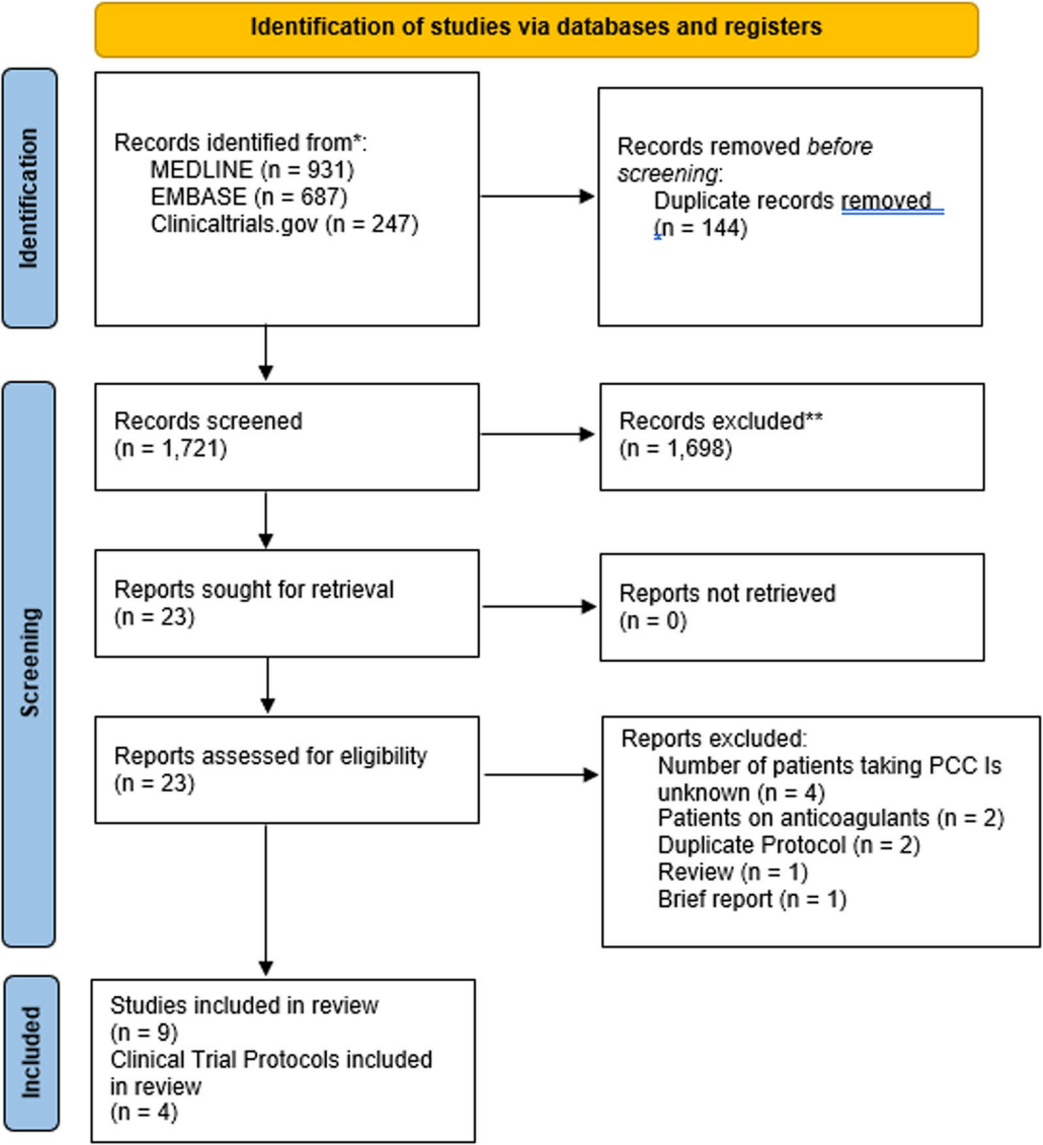
PRISMA flow diagram search strategy [24]

Following the search strategy, 1925 publications were identified (MEDLINE: 942, EMBASE: 727, ClinicalTrials.gov: 256). After removal of 180 duplicates, 1745 papers were screened and 1722 of these were excluded according to eligibility criteria. Full-text review was applied to the remaining 23 studies, and of these, nine observational studies [22–30], four clinical trial protocols [31–34], and one randomized control trial were included in the analysis [35]. The PRISMA flow diagram can be found in Fig. 1.

### Observational studies

#### Characteristics of the studies and of their patients

Across all nine observational studies, a total of 823 patients were included in the PCC treatment group (ranging from 9 to 234 patients per group). Two of the studies were multi-center with the remainder being single center [25, 27]. The mean a^26^ge of the patients ranged from 36 to 51 years and the mean ISS from 23 to 50. Blunt mechanisms predominated and penetrating injuries comprised 13% to 23% of the patients receiving PCC. Only four studies reported INR at admission, and the mean values ranged from 1.8 to 2.3 [22–24, 26]. Three studies reported pre-injury antiplatelet use, with proportions ranging from 19 to 27% [22, 25, ]. The main characteristics of the included studies can be found in Table 1.

**Table 1:** Characteristics of included observational studies

First author Year Country	Study design	Age in the PCC group(s)	Sex in the PCC group(s)	ISS in the PCC group(s)	INR in the PCC group(s)	Penetrating injuries in the PCC group(s)	PCC Indication	Number of patients in the PCC group^&^	Intervention Group 1	Intervention Group 2	Control Group
Jehan [26] US	Single center with PS matching	57 (20.9)^#&^	26 (65)^&^	30 [21–38]^%&^	1.8 (2)^#^	7 (17)	Significant bleeding and coagulopathic (INR ≥ 1.5)	*n* = 40	4F-PCC (25 units/kg) + FFP^£^	–	FFP alone^£^
Joseph [31] US	Single center with PS matching	46 (22)^#^	49 (78)	28 [17–40]^%&^	2.2 (0.9)^#^	14 (23)	At the discretion of the attending trauma surgeon	*n* = 63	3F-PCC (25U/kg) + FFP (15 mL/Kg)	–	FFP alone (15 mL/Kg)
Joseph [31 US	Single center with PS matching	48.3 (23.2)^#^	20 (74)	24 (14–31)^%!^	2.3 (0.8)^#^	6 (22)	At the discretion of the attending trauma surgeon	*n* = 27	3F-PCC (25U/kg)	–	FFP alone (15 mL/Kg)
Khurrum [27 US	Multicenter with PS matching	48 (21)^#&^	102 (61)^&^	30 [21–38]^%&^	–	11 (13)	Unknown	*n* = 84	4F-PCC^£^ + Whole Blood^£^	–	Whole Blood
Ponschab [30] Austria	Single center without adjustment	45 [26.3–60.0]^%^	69 (71.9)	Unknown	–	–	EXTEM > 80 s after FC treatment OR Obvious severe coagulopathy OR Physician discretion	PPC alone: *n* = 13 PCC + FC: *n* = 23	PCC alone^£^^	FC (4 g) + PCC^£^^	FC alone (4 g)
Schlimp [28 Austria	Single center without adjustment	FC-PCC: 45 [26–57]^%^ FC-PCC-FFP: 49 [29–58]^%^	FC-PCC: 51 (81) FC-PCC-FFP: 6 (67)	FC–PCC: 34 [26–43]^%^ FC–PCC–FFP: 50 [42–58]^%^	–	–	EXTEM > 80 s after FC treatment	FC + PCC: n = 63 FC + PCC + FFP: n = 9	FC (2 to 6 g) + 4F-PCC (20 to 30 IU/kg)	FC alone (2 to 6 g)	Fibrinogen concentrate (2-6 g) + 4F-PCC (20 to 30 IU/kg) + FFP^£^
Schöchl [29] Austria	Single center without adjustment	FC: 40 (14)^#^ FC + PCC: 36 (13)^#^	NA	35.7 (13.0)^#^	–	–	EXTEM > 80 s after FC treatment OR Obvious severe coagulopathy OR Physician discretion	*n* = 17	FC (6-8 g) + 4F-PCC (20 to 30 IU/kg)	FC alone (6-8 g)	No coagulation therapy
Zeeshan [22] US	Single center with PS matching	4F-PCC: 51 (19.6)^#^ 3F-PCC: 50 (18.3)^#^	4F-PCC: 82 (66) 3F-PCC: 80 (64)	4F-PCC: 23 [14–32]^%&^ 3F-PCC: 27[15–31]^%&^	4F-PCC: 2.0 (0.3)^#&^ 3F-PCC: 1.9 (0.2)^#&^	4F-PCC: 21 (17)^&^ 3F-PCC: 18 (14)^&^	Trauma-induced coagulopathy with INR ≥ 1.5	4F-PCC: *n* = 125 3F-PCC: *n* = 125	4F-PCC (25U/kg) + FFP^£^	–	3F-PCC (25U/kg) + FFP^£^
Zeeshan [25] US	Multi center with PS matching	50 (21)^#&^	171 (70.3)^&^	27 [20–37]^%&^	–	32 (13.4)	Unknown	*n* = 234	4F-PCC^£^ + FFP^£^	–	FFP alone^£^

Continuous variables are presented either as median (IQR) ^%^ or as mean (SD)^#^; categorical variables are presented as number and relative percentages. Number of patients or relative percentages were calculated if not reported. Some variables are presented after PS matching^&^ while other are presented before PS matching^!^. ^£^ Protocol doses are unknown; ^Unknown type of PCC. *FC* Fibrinogen concentrate, *ISS* injury severity score, *INR* international normalized ratio, *PCC* prothrombin complex concentrate, *PS* propensity score, *US* United States

#### PCC administration

The most commonly compared type of prothrombin complex concentrate was 4F-PCC. Overall, six studies evaluated 4F-PCC [22, 25–29], three studies used 3F-PCC [22–24], one did not report the type of PCC used [30], and one study compared 3F-PCC and 4F-PCC [22]. The specific pharmaceutical products used for 3F-PCC treatment were Profilnine SD**®** (Grifols, Los Angeles, Calif) in three studies [22–24] or Bebulin VH**®** (Baxter Healthcare Corporation, Deerfield, Ill) in one study [22]). The product used for 4F-PCC treatment was either Prothromplex**®** (Baxter, Vienna, Austria) (*n* = 2) [28, 29] or Kcentra**®** (CSL Behring, Germany) (*n* = 2) [22, 26]. Two studies did not specify the product used [25, 27].

The recommended dose of FFP in each study ranged from 20 to 30 mL/kg, but the dose which was actually administered was not reported [22–24, 26, 28, 29]. The mean time of PCC administration was reported in four studies and ranged from 26 to 68 min after admission  [22–25]. Indications for PCC administration included TIC with an INR ≥ 1.5 (*n* = 2)  [22, 26], at the discretion of the attending physician (*n* = 3) [23, 24, 29], if EXTEM was superior to 80 s after fibrinogen concentrate administration (*n* = 3) [28–30] or unknown (*n* = 2) [25, 27]. None of the studies reported whether treatments were administered in accordance with protocols or guidelines.

#### Co-interventions

PCC was used as stand-alone coagulation treatment in two studies [23, 30], while in the others PCC was used in association with supplementary treatments such as FFP (*n* = 4) [22, 23, 25, 26], fibrinogen concentrate (*n* = 2) [28, 29], or whole blood (*n* = 1) [27]. Four studies reported the proportion of patients receiving tranexamic acid [22, 26, 28, 30]. Patients requiring surgical procedures were inconsistently reported among the studies, and none stated the proportion of patients who received damage control surgery.

Comparators The most frequent comparator of PCC was FFP, either administered alone (*n* = 4)  [23–26], associated with another treatment such as another type of PCC (*n* = 1) [22] or with PCC and fibrinogen concentrate (*n* = 1) [28]. There was one comparison of whole blood with and without PCC [27], and three studies used fibrinogen concentrate as a comparator [28–30]. No coagulation therapy was used as a comparator in a single study [29].

#### Outcomes

In-hospital mortality was an outcome in eight studies and emergency department (ED) mortality in two (Table 2). Among six propensity-matched studies, four compared PCC to FPP alone [23–26], and three of these reported a significantly lower mortality with PCC  [23, 25, 26]. Conversely, Joseph et al*.* [24] did not find any mortality difference using the same comparison (6% vs 15%, *p* = 0.78). When PCC was added to whole blood, Khurrum et al*.* reported no effect on either ED mortality (6% vs 4%; *p* = 0.42) or in-hospital mortality (44% vs 46%; *p* = 0.72) [27]. Similarly, there were no significant reductions in mortality when 3F-PCC was compared to 4F-PCC, both with FFP (32%, vs 35%; *p* = 0.78) [22]. Schlimp et al*.* reported, for those only receiving fibrinogen concentrate and in a non-adjusted comparison, the lowest mortality followed by combinations of fibrinogen concentrate + PCC, and fibrinogen concentrate + PCC + FFP (respectively, 8%, 29%, and 56%; *p* < 0.001) [28].

**Table 2:** Outcomes reported by included studies

Studies references	In-hospital Mortality	DVT In hosp	RBC (unit)	Platelets (unit)	FFP (unit)	Other outcomes reported
Jehan [26]	PCC + FFP: 10 (25) FFP 26 (33) *p* = 0.04*	PCC + FFP: 1 (2.5) FFP: 1 (1.2) *p* = 0.51*	*Unclear* PCC + FFP: 7 (3)^µ^ FFP: 9 (5)^µ^ *p* < 0.04*	*Unclear* PCC + FFP: 3 (3)^µ^ FFP: 3 (3)^µ^ *p* = 0.72*	*Unclear* PCC + FFP: 5 (2)^µ^ FFP: 7 (3)^µ^ *p* = 0.03*	Proportion of patients with INR correction and time to INR correction from admission* Rate of correction of INR* Other TE*: PE, Mesenteric ischemia ICU and Hospital LOS
Joseph [31]	PCC + FFP: 15 (23) FFP alone: 53 (28) *p* = 0.04	PCC + FFP: 1 (1.6) FFP alone: 2 (1.1) *p* = 0.6*	*Total* PCC + FFP: 6.6 (4.1)^µ^ FFP alone: 10 (8.3)^µ^ *p* = 0.001*	*Total* PCC + FFP: 1.2 (2.1)^µ^ FFP alone: 1.5 (2.7)^µ^ *p* = 0.2*	*Total* PCC + FFP: 2.8 (1.8)^µ^ FFP alone: 3.9 (1.3)^µ^ *p* = 0.01*	Proportion of patients with INR correction and time to INR correction from admission* Time to treatment* Mesenteric infarction* ICU and Hospital LOS Costs: therapy*, transfusion*, hospital
Joseph [24]	PCC: 6 (22.3) FFP 15 (27.8) *p* = 0.78	PCC: 3 (11.1) FFP: 4 (7.4) *p* = 0.68*	*Total* PCC: 3.2 (1.9)^µ^ FFP: 5.4 (4.1)^µ^ *p* = 0.009*	*Total* PCC: 1.4 (2.3)^µ^ FFP: 1.6 (2.4)^µ^ *p* = 0.72*	*Total* PCC: 5.1 (3.6)^µ^ FFP: 90.7 (4.1)^µ^ *p* = 0.005*	Proportion of patients with INR correction and time to INR correction from admission* Time to treatment Time to surgery Mesenteric or myocardial infarction ICU and Hospital LOS Costs: therapy, transfusion, hospital, charges
Khurrum [27]	PCC + WB: 39 (46) WB 74 (44) *p* = 0.72*	PCC + WB: 3 (4) WB: 8 (5) *p* = 0.75	*At 24 h* PCC + WB: 8 (5–14)^$^ WB: 10 (6–18)^$^ *p* = 0.04*	*At 24 h* PCC + WB: 2 (1–3)^$^ WB: 2 (1–4)^$^ *p* = 0.19*	*At 24 h* PCC + WB: 6 (4–10)^$^ WB: 8 (4–12)^$^ *p* = 0.01*	PCC units In ED mortality ED, ICU and hospital LOS AKI, ARDS, PE
Ponschab [30]	–	–	*At 24 h* All patients: 7 (3–10) [7]	*At 24 h* All patients: 0 (0–00) [7]	*At 24 h* All patients: 0 (0–0)^7^	ROTEM parameter PCC unit, FC (g) and TXA (g) administrated at 24 h
Schlimp [28]	PCC + FC: 18 (29) FC: 7 (8) PCC + FC + FFP: 5 (56) p < 0.0001	–	*At 24 h* PCC + FC: 8 (5–11)^$^ FC: 3 (2–6)^$^ PCC + FC + FFP: 21 (18–26)^$^ *p* < 0.0001	*At 24 h* PCC + FC: 0 (0–0)^$^ FC: 0 (0–0)^$^ PCC + FC + FFP: 4 (2–4)^$^ *p* < 0.001	*At 24 h* PCC + FC: none FC: none PCC + FC + FFP: 6 (6–10)^$^ *p* = NA	Transfusion (RBC, FFP, platelet, FC, PCC) volume in ED and ICU Massive transfusion Standard and specific coagulation tests including ROTEM over 7 days
Schöchl [29]	PCC + FC: 0 (0) FC: 0 (0) NCT: 0 (0)	-	*At 24 h* PCC + FC: 8 (6–10.5)^$^ FC: 3 (0–5)^$^ NCT: 0 (0–2)^$^ *p* < 0.001	At 24 h PCC + FC: 0 (0–1)^$^ FC: 0 (0–0)^$^ NCT: 0 (0–0)^$^ *p* < 0.001	*At 24 h* PCC + FC: 0 (0–0)^$^ FC: 0 (0–0)^$^ NCT: 0 (0–0)^$^ *p* = ns	PCC and FC unit transfused Standard and specific coagulation tests including ROTEM over 7 days
Zeeshan [25]	4F-PCC: 32 (26) 3F-PCC: 35 (28) *p* = 0.78*	4F-PCC: 2 (1.4) 3F-PCC: 3 (2.1) *p* = 0.81*	*Total* 4F-PCC: 7 (2)^µ^ 3F-PCC: 10 (3)^µ^ *p* = 0.04*	*Total* 4F-PCC: 3 (3)^µ^ 3F-PCC: 3 (3)^µ^ *p* = 0.23*	*Total* 4F-PCC: 6 (2)^µ^ 3F-PCC: 8 (2)^µ^ *p* = 0.03*	Proportion of patients with INR correction and time to INR correction from admission* ICU and Hospital LOS Other TE*: Mesenteric infarction, PE Costs: therapy, transfusion, total hospital
Zeeshan 2018 [26]	4-PCC + FFP: 41 (17.5) FFP: 65 (27.7) *p* = 0.01*	PCC + FFP: 8 (3.4) FFP: 13 (5.5) *p* = 0.11	*At 24 h* PCC + FFP: 6 (4)^µ^ FFP: 10 (4)^µ^ *p* = 0.02*	*At 24 h* PCC + FFP: 3 (2)^µ^ FFP: 3 (3)^µ^ *p* = 0.72*	*At 24 h* PCC + FFP: 3 (2)^µ^ FFP: 6 (3)^µ^ *p* = 0.01*	ED mortality* Transfusion at 4 h* ICU and Hospital LOS Skilled nursing facility or rehab. disposition Complications: AKI, ARDS, PE

* Continuous variables are presented either as median (IQR) ^%^ or as mean (SD)^#^; categorical variables are presented as number and relative percentages. Number of patients or relative percentages were calculated if not reported. * Reported as primary outcome(s). Some variables are presented after PS matching^&^ while others are presented before PS matching^!^. ^£^ Protocol doses are unknown; ^Unknown type of PCC. *AKI* Acute Kidney Injury, *ARDS* acute respiratory distress syndrome, *ED* emergency department, *FC* fibrinogen concentrate, *ICU* intensive care unit, *ISS* injury severity score, *INR* international normalized ratio, *LOS* length of stay, *PE* pulmonary embolism, *PCC* prothrombin complex concentrate, *PS* propensity score, *TXA* tranexamic acid, *US* United States

The overall pooled odds ratio (OR) of in-hospital mortality across all observational studies was 0.97 [95% CI 0.56–1.69] with a high proportion of heterogeneity (I^2^ = 70%) (Additional file 1). In propensity-matched studies the pooled OR was 0.75 [95% CI 0.54–1.04] with low heterogeneity (I^2^ = 0%), while one non-adjusted study reported events with an OR of 4.46 [95% CI 1.73–11.49] (Additional file 1).

Five studies reported DVT incidence which ranged from 1.6 to 11.1% in groups treated by PCC + FFP and PCC alone [23–27] (Table 2). There was no difference in DVT incidence when comparing 4F-PCC + FFP with 3F-PCC + FFP groups (2.1% vs 1.4%; *p* = 0.81) [22]. The pooled odds ratio of DVT was 0.83 [95% CI 0.44–1.57] with no heterogeneity (I^2^ = 0%) (Additional file 1). PCC administration was also not associated with pulmonary embolism in three studies [25–27], and when comparing the 3F-PCC + FFP group and the 4F-PCC + FFP group, no change in the incidence of PE was reported [25].

All studies reported transfusion volume (RBC, FFP, and platelets) either at hospital discharge or at 24 h. Seven compared a group with PCC to a group without PCC irrespective of the co-treatment involved [23, 24, 26–29]. Five of these seven studies used propensity score (PS) adjustment and reported a significant reduction in RBC and FFP use [23–27], but platelet transfusions were not reduced by PCC. At 24 h, Zeeshan et al. reported a reduction from 10 to 6 units of RBC when comparing PCC alone to FPP alone (*p* = 0.02) [25]. Similarly, Khurrum et al. reported a reduction from 10 to 8 units of RBC when comparing PCC with whole blood to whole blood alone (*p* = 0.04) [27] (Table 2).

Two studies did not adjusted comparisons and observed that patients treated with PCC received significantly more RBC and platelets, but not FFP [28, 29]. When comparing 3F-PCC + FFP to 4F-PCC + FFP, the 4F-PCC + FFP intervention group had a reduced average number of RBC requirements (10 RBC units vs 7 RBC units; *p* = 0.04) and the FFP requirements (8 FFP units vs 6 FFP units; *p* = 0.03), while platelet requirements were not altered (3 vs 3 units; *p* = 0.23) (Table 2) ^(1)^. The variation in reporting time points of the transfusion volumes (either 24 h, overall, or unclear) prevented meta-analysis for these outcomes.

#### Quality assessment

**Fig. 2:**
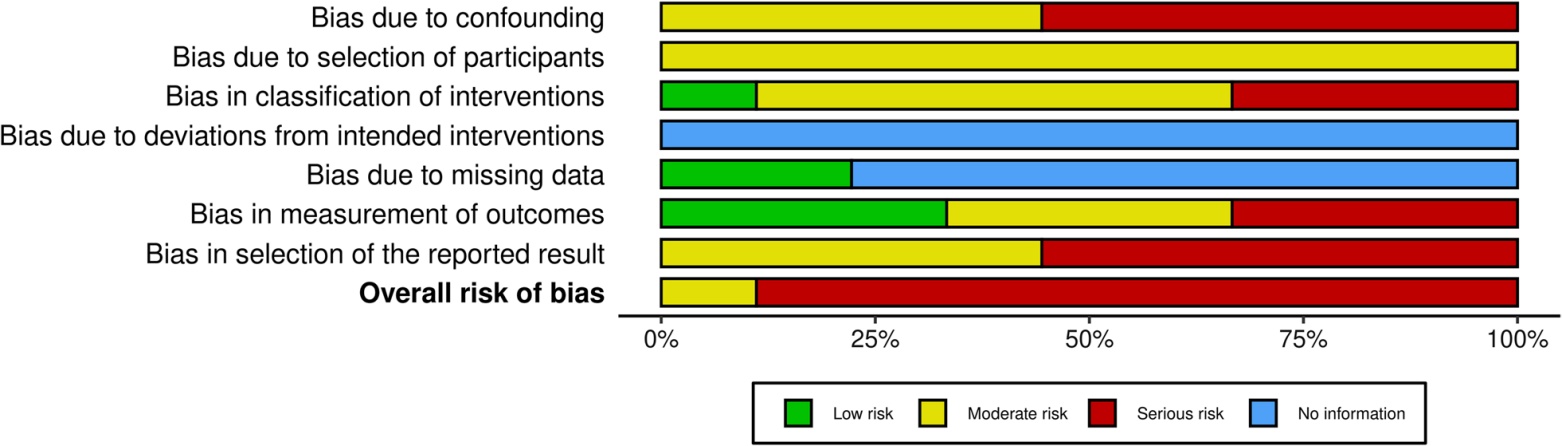
Risk of bias summary plot [25]

The overall risk of bias was serious in eight studies [23–30] and moderate in one study [22] (Fig. 2, and Additional file 1**)**. Bias due to “deviations from intended interventions” and to “missing data” could not be properly assessed due to the lack of information available in the majority of the manuscripts.

### Published randomized clinical trial

PROCOAG was the only RCT identified. In this double-blind, placebo-controlled (saline solution) superiority trial, 327 patients at risk of massive transfusion were recruited in 12 French trauma centers to empirically receive 4F-PCC (25 U/Kg) in addition to a ratio-based massive transfusion protocol including fibrinogen concentrate [35]. There was no significant between-group difference for the primary outcome with a median 24-h total number of blood products of 12 [5–19] in the 4F-PCC group versus 11 [6–19] in the placebo group (*p* = 0.72). The trial nevertheless highlighted that 56 patients (35%) presented with at least one thromboembolic event in the 4F-PCC group compared to 37 (24%) in the placebo group (*p* = 0.03). PCC administration had no effect on the 28-day mortality, with 17% (*n* = 26) dying in the PCC group and 21% (*n* = 30) in the placebo group (*p* = 0.48).

**Fig. 3:**
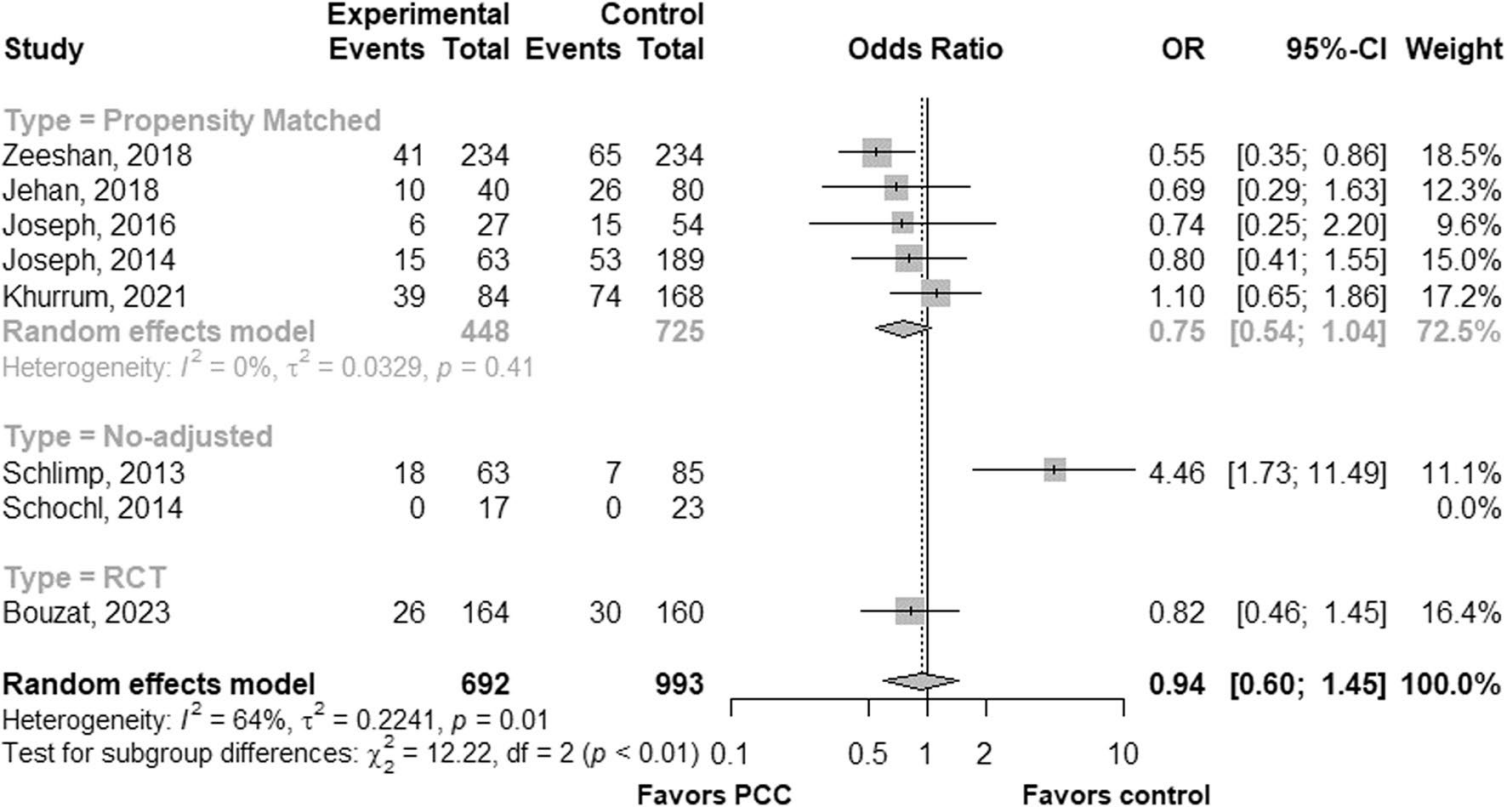
In-hospital or 28-day mortality forest plot

**Fig. 4:**
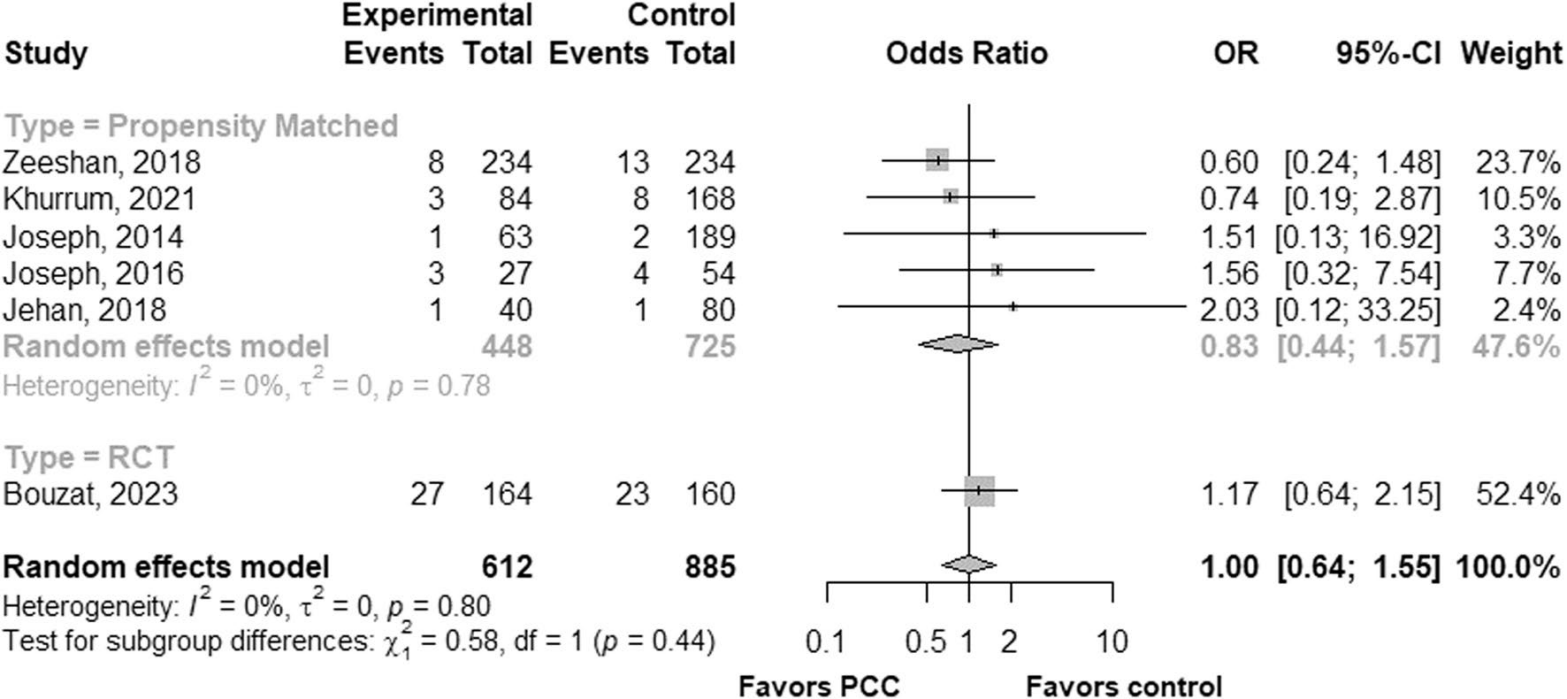
In-hospital deep venous thrombosis forest plot

When the results from observational studies were pooled with the findings from this RCT, the OR of 0.94 [95% CI 0.60–1.45] confirmed that PCC given to treat TIC had no effect on mortality (Fig. 3, Additional file 1). The pooled odds ratio (OR) for DVT, combining results from both randomized controlled trials (RCTs) and observational studies, was 1.00 (95% CI 0.64–1.55). (Fig. 4). However, it was not possible to meta-analyze the risk of having at least one thromboembolic event as this outcome was not reported in the observational studies. The risk of bias, evaluated using the ROB2 tool, was low in this trial (Additional file 1).

### Ongoing clinical trials

We identified four ongoing RCTs involving the use of PCC in adult trauma patients **(**Additional file 1**)**. The “*REPLACE*” (Randomized Trial Evaluating the Use of Prothrombin complex concentrate to Improve Survival in Patients With Traumatic Coagulopathy, NCT03981484) [31], the “*FiiRST-2*” (Factor In the Initial Resuscitation of Severe Trauma 2, NCT04534751) trial [32], “Prehospital Use of 4F-PCC for Hemorrhagic Shock trial” (NCT04019015) [33], and the “Evaluation of the Efficacy of Early Bunching of a FF-PCC in Patients With Severe Traumatic Hemorrhage” trial (NCT05738642) [34]. None of these trials reported results yet.

### Quality of evidence according to the Grading of Recommendation Assessment, Development, and Evaluation (GRADE)

The summary of the quality of evidence according to the Grading of Recommendation Assessment, Development, and Evaluation (GRADE) is reported in Table 3.

**Table 3:** Summary of the quality of evidence according to the Grading of Recommendation Assessment, Development, and Evaluation (GRADE)

Patient or population: Adult patients with expected trauma-induced coagulopathy Settings: In-hospital care Intervention: Patients treated with PCC Comparison: Patients treated without PCC	

Outcomes No of participants (studies)	Odds ratio	Anticipated absolute effects			Certainty of the evidence (GRADE)	Comments
		Without PCC	With PCC	Difference		
Mortality (8 studies, n = 1685)	0.94 [0.60, 1.45]	27.2%	22.4%	− 4.8%	⊕ ⊝ ⊝ ⊝ Very low^a,b^	May decrease in-hospital mortality
DVT (6 studies, n = 1497)	1.00 [0.64; 1.55]	5.3%	7.0%	+ 1.3%	⊕ ⊝ ⊝ ⊝ Very Low^a,c,d^	May increase DVT

^a^Downgraded because the overall risk of bias for observational studies was serious

^b^Downgraded because PCC administration was not associated with mortality reduction in the only RCT published

^c^Downgraded because of the large confidence intervals

^d^Downgraded because the only RCT did not reported a significant increase of the DVT (but an increase of a composite outcome regrouping the occurrence of at least one thromboembolic event)

*DVT* deep venous thrombosis, *PCC* prothrombin complex concentrate

## Discussion

This meta-analyses evaluated the effects of PCC administration in trauma patients with TIC and demonstrated that amongst eight observational studies and one RCT, PCCs were not associated with a mortality reduction. Pooled data in observational studies did not reveal an increased VTE risk. However, the only RCT found reported increased rate of thromboembolic events in patients allocated to the PCC group. In addition, the qualitative analysis demonstrated the low level of evidence on which the use of PCCs to treat TIC currently relies. Most included studies are non-randomized, have serious risk of bias with inconsistent or no adjustment methods, and small numbers of patients from single centers.

We found no overall beneficial effect of PCC use on in-hospital mortality although four studies included reported statistically significant reductions [23, 25, 26, 28]. Three of these studies involved the use of FFP as co-treatments of PCC, which may have contributed to the observed treatment effect on mortality [23, 25, 26]. Of note, four of the six propensity-matched studies were published by the same research team, using similar datasets [23–26], and three of these four studies were included in the meta-analysis by Kao et al. [36]. These findings may suggest a potential publication bias, which has, at the end, influenced the European guideline on management of major bleeding [10].

Incidence of VTE was 35% in the PCC treatment arm compared to placebo (24%) in the RCT. Other studies have found rates of VTE from 3 to 15% following routine screening. [12]. TIC is comprised of several interconnected phenotypes attributed to differing mechanistic responses occurring at varying time points post-injury [37]. Procoagulant treatments may be administered during the hypocoagulant phase of TIC which may produce effects that influence or strengthen subsequent hypercoagulability leading to increased VTE. The recent PROCOAG RCT emphasized this possibility, where it is likely that patients without thrombin generation deficit received PCC, exposing them to a thrombotic risk, while they were unlikely to benefit from the intervention [38]. This highlights the need in current practice for targeted procoagulant treatments where VTE risk screening and side effect assessment are provided alongside PCC administration. This also provides the impetus for future research to determine whether there are patient phenotypes for which the administration of PCC can both improve survival while avoiding an excessive risk of VTE.

All four ongoing RCTs will empirically administer PCC, based on clinical observations, such as blood pressure or evidence of active bleeding, or on physicians' expectations, such as predicted red blood cell transfusion. Moreover, only one of them will include VTE as an outcome [32]. Consequently, these RCTs will not be able to direct treatment administration based on the presence of a phenotype most likely to benefit from PCC with the lowest risk of harm. This may reduce these studies capacity to delineate the risk–benefit balance of PCC administration and may warrant further investigations.

### Limitations

This systematic review has several limitations. Firstly, trauma patients with hemorrhage are complex, frequently requiring a broad range of interventions. Therefore, identifying the precise treatment effect and/or side effects of a given intervention, such as using PCC, poses a challenge, as evidenced by the multiple inconclusive RCTs published in trauma research [35, 39–42]. Furthermore, as a result of this underlying complexity, studies included in this systematic review reported on different populations, indications, ways to administrate PCCs, co-treatments, and comparators.

Second, the lack of standardization in reporting of outcomes was a concern. For instance, mortality and transfusion volumes were reported at various time points, including the emergency department, 24 h, intensive care units, hospital discharge, or at 30 days. Similarly, only six observational studies reported on the occurrence of DVT which may under-represent the incidence.

Third, we used mortality at discharge as a primary outcome, which is known to be a challenging outcome to be measure in studies enrolling coagulopathic trauma patients. For these reasons, it might have been worthwhile to consider other outcomes, such as the correction of coagulopathy, the volume of allogeneic blood products transfused, or earlier mortality. As an example, the PROCOAG study recently employed the median 24-h total number of blood products as a primary outcome.

Finally, among the included studies, PCC was administrated either at the discretion of the attending physician, based on the clinical severity of the patient, or guided by laboratory results. It is not certain that both of these methods are sufficient to accurately identify patients who are truly likely to benefit from PCC treatment. Clinically determining hemorrhage at an early stage is challenging, while scoring systems exist for bleeding and coagulopathy, they lack sensitivity [43]. Treatment efficacy is indeed constantly modulated by a range of variables such as the patients baseline characteristics or the effects of co-interventions and the assessment of traumatic hemorrhage has relied on a combination of factors including clinical, physiological, and imaging parameters [10].

## Conclusion

This systematic review exposes the current heterogeneity associated with PCC administration during trauma hemorrhage. It also highlights that among included studies, PCC did not improve in-hospital mortality, nor it is reported to increase VTE. The results of this systematic underpin the urgent need for further high-level studies to determine PCC efficacy, safety, and indications among patient with TIC.

### Supplementary Information

**Additional file 1.** Online Supplementary Materials.

## Data Availability

The datasets used and/or analyzed during the current study are available from the corresponding author on reasonable request.
