# Supplementary material for: Prothrombin complex concentrate (PCC) for treatment of trauma-induced coagulopathy: systematic review and meta-analyses
Source: Crit Care. 2023 Nov 2;27:422. doi: 10.1186/s13054-023-04688-z (PMC10621181; doi:10.1186/s13054-023-04688-z)
Supplement: Supplementary file 1 — Additional file 1. Online Supplementary Materials. [file 13054_2023_4688_MOESM1_ESM.docx]

# Supplementary material

## Supplementary material 1: PRISMA checklist

| **Section and Topic** | **Item #** | **Checklist item** | **Location where item is reported** |
| --- | --- | --- | --- |
| **TITLE** | | |  |
| Title | 1 | Identify the report as a systematic review. | Title |
| **ABSTRACT** | | |  |
| Abstract | 2 | See the PRISMA 2020 for Abstracts checklist. | Appendix 1 |
| **INTRODUCTION** | | |  |
| Rationale | 3 | Describe the rationale for the review in the context of existing knowledge. | Intro |
| Objectives | 4 | Provide an explicit statement of the objective(s) or question(s) the review addresses. | Intro |
| **METHODS** | | |  |
| Eligibility criteria | 5 | Specify the inclusion and exclusion criteria for the review and how studies were grouped for the syntheses. | Inclusion & Exclusion criteria |
| Information sources | 6 | Specify all databases, registers, websites, organisations, reference lists and other sources searched or consulted to identify studies. Specify the date when each source was last searched or consulted. | Data source & strategy |
| Search strategy | 7 | Present the full search strategies for all databases, registers, and websites, including any filters and limits used. | Appendix 2 |
| Selection process | 8 | Specify the methods used to decide whether a study met the inclusion criteria of the review, including how many reviewers screened each record and each report retrieved, whether they worked independently, and if applicable, details of automation tools used in the process. | Selection of studies |
| Data collection process | 9 | Specify the methods used to collect data from reports, including how many reviewers collected data from each report, whether they worked independently, any processes for obtaining or confirming data from study investigators, and if applicable, details of automation tools used in the process. | Data extraction |
| Data items | 10a | List and define all outcomes for which data were sought. Specify whether all results that were compatible with each outcome domain in each study were sought (e.g. for all measures, time points, analyses), and if not, the methods used to decide which results to collect. | Data extraction |
|  | 10b | List and define all other variables for which data were sought (e.g. participant and intervention characteristics, funding sources). Describe any assumptions made about any missing or unclear information. | Statistical Analysis |
| Study risk of bias assessment | 11 | Specify the methods used to assess risk of bias in the included studies, including details of the tool(s) used, how many reviewers assessed each study and whether they worked independently, and if applicable, details of automation tools used in the process. | Risk of bias assessment |
| Effect measures | 12 | Specify for each outcome the effect measure(s) (e.g. risk ratio, mean difference) used in the synthesis or presentation of results. | Statistical Analysis |
| Synthesis methods | 13a | Describe the processes used to decide which studies were eligible for each synthesis (e.g. tabulating the study intervention characteristics and comparing against the planned groups for each synthesis (item #5)). | Data extraction |
|  | 13b | Describe any methods required to prepare the data for presentation or synthesis, such as handling of missing summary statistics, or data conversions. | Statistical Analysis |
|  | 13c | Describe any methods used to tabulate or visually display results of individual studies and syntheses. | Statistical Analysis |
|  | 13d | Describe any methods used to synthesize results and provide a rationale for the choice(s). If meta-analysis was performed, describe the model(s), method(s) to identify the presence and extent of statistical heterogeneity, and software package(s) used. | Statistical Analysis |
|  | 13e | Describe any methods used to explore possible causes of heterogeneity among study results (e.g. subgroup analysis, meta-regression). | Statistical Analysis |
|  | 13f | Describe any sensitivity analyses conducted to assess robustness of the synthesized results. | 0 |
| Reporting bias assessment | 14 | Describe any methods used to assess risk of bias due to missing results in a synthesis (arising from reporting biases). | Risk of bias assessment |
| Certainty assessment | 15 | Describe any methods used to assess certainty (or confidence) in the body of evidence for an outcome. |  |
| **RESULTS** | | |  |
| Study selection | 16a | Describe the results of the search and selection process, from the number of records identified in the search to the number of studies included in the review, ideally using a flow diagram. | Figure 1 |
|  | 16b | Cite studies that might appear to meet the inclusion criteria, but which were excluded, and explain why they were excluded. | Search results |
| Study characteristics | 17 | Cite each included study and present its characteristics. | Appendix 3 |
| Risk of bias in studies | 18 | Present assessments of risk of bias for each included study. | Figure 5 |
| Results of individual studies | 19 | For all outcomes, present, for each study: (a) summary statistics for each group (where appropriate) and (b) an effect estimate and its precision (e.g. confidence/credible interval), ideally using structured tables or plots. | Figure 2 & 3 |
| Results of syntheses | 20a | For each synthesis, briefly summarise the characteristics and risk of bias among contributing studies. | Risk of bias |
|  | 20b | Present results of all statistical syntheses conducted. If meta-analysis was done, present for each the summary estimate and its precision (e.g. confidence/credible interval) and measures of statistical heterogeneity. If comparing groups, describe the direction of the effect. | Result |
|  | 20c | Present results of all investigations of possible causes of heterogeneity among study results. | Discussion |
|  | 20d | Present results of all sensitivity analyses conducted to assess the robustness of the synthesized results. | Risk of bias |
| Reporting biases | 21 | Present assessments of risk of bias due to missing results (arising from reporting biases) for each synthesis assessed. | Not applicable |
| Certainty of evidence | 22 | Present assessments of certainty (or confidence) in the body of evidence for each outcome assessed. | Not applicable |
| **DISCUSSION** | | |  |
| Discussion | 23a | Provide a general interpretation of the results in the context of other evidence. | Discussion with ongoing litterature |
|  | 23b | Discuss any limitations of the evidence included in the review. | Limitations |
|  | 23c | Discuss any limitations of the review processes used. | Limitations |
|  | 23d | Discuss implications of the results for practice, policy, and future research. | Implications |
| **OTHER INFORMATION** | | |  |
| Registration and protocol | 24a | Provide registration information for the review, including register name and registration number, or state that the review was not registered. | Not registered |
|  | 24b | Indicate where the review protocol can be accessed, or state that a protocol was not prepared. | 0 |
|  | 24c | Describe and explain any amendments to information provided at registration or in the protocol. | 0 |
| Support | 25 | Describe sources of financial or non-financial support for the review, and the role of the funders or sponsors in the review. | Acknowledgement section |
| Competing interests | 26 | Declare any competing interests of review authors. | Acknowledgement section |
| Availability of data, code and other materials | 27 | Report which of the following are publicly available and where they can be found: template data collection forms; data extracted from included studies; data used for all analyses; analytic code; any other materials used in the review. | Acknowledgement section |

*From:*  Page MJ, McKenzie JE, Bossuyt PM, Boutron I, Hoffmann TC, Mulrow CD, et al. The PRISMA 2020 statement: an updated guideline for reporting systematic reviews. BMJ 2021;372:n71. doi: 10.1136/bmj.n71

## Supplementary Material 2: Search equations

### Search equation (MEDLINE)

#Population#

1. severe trauma[tiab]
2. severe injur*[tiab]
3. poly trauma[tiab]
4. polytrauma[tiab]
5. multiple wound*[tiab]
6. multiple injur*[tiab]
7. multiple trauma[tiab]
8. major trauma[tiab]
9. major injur*[tiab]
10. multiple trauma [MeSH Terms]
11. critical bleed*[tiab]
12. trauma induced coagulopathy [tiab]
13. TIC[tiab]
14. trauma [tiab]
15. post trauma haemorrhage[tiab]
16. post trauma hemorrhage[tiab]
17. #1 OR #2 OR #3 OR #4 OR #5 OR #6 OR #7 OR #8 OR #9 OR #10 …..

**295,967 results**

#Intervention#

1. Prothrombin complex concentrates[tiab]
2. PCC[tiab]
3. Cofact[tiab]
4. Beriplex[tiab]
5. Confidex[tiab]
6. Octaplex[tiab]
7. Kcentra[tiab]
8. PPSB[tiab]
9. PPSB SD[tiab]
10. Prothrombinex-VF[tiab]
11. Prothrombinex[tiab]
12. prothrombin complex[tiab]
13. blood coagulation factors[MeSH Terms]
14. blood Coagulation Disorders[MeSH Terms]
15. blood coagulation factors[tiab]
16. blood[tiab] AND coagulation[tiab] AND factors[tiab]
17. 4-factor[tiab] OR four-factor[tiab] OR 4F-PCC[tiab]OR 4-PCC[tiab] OR PCC4[tiab]
18. 3-Factor[tiab] OR three-factor[tiab] OR 3F-PCC[tiab]OR 3-PCC[tiab] OR PCC3[tiab]
19. OR #18 OR #19 OR #20 …..

**567,914 results**

#Adults#

1. adult[mh] NOT child[mh] NOT adolescent [mh] NOT infant[mh]

#2010 – 2022#

1. "2010/01/01"[Date - Publication] : "2022/11/01"[Date - Publication]

#Total#

1. #17 AND #36 AND #37 AND #38

**931 results**

### Search equation (EMBASE)

#Population

1. 'severe trauma':ab,ti
2. 'severe injur*':ab,ti
3. 'poly trauma':ab,ti
4. polytrauma:ab,ti
5. 'multiple wound*':ab,ti
6. 'multiple injur*':ab,ti
7. multiple trauma':ab,ti
8. 'major trauma':ab,ti
9. 'major injur*':ab,ti
10. 'multiple trauma'/exp
11. 'critical bleed*':ab,ti
12. 'trauma induced coagulopathy':ab,ti
13. tic:ab,ti
14. 'post trauma haemorrhage':ab,ti
15. 'post trauma hemorrhage':ab,ti
16. 'massive transfusion':ab,ti
17. coagulopathy:ab,ti
18. #1 OR #2 OR #3 OR #4 OR #5 OR #6 OR #7 OR #8 OR #9 OR #10 OR #11 OR #12 OR #13 OR #14 OR #15 OR #16 OR #17

**84,057 results**

#Interventions

1. 'prothrombin complex concentrates':ab,ti
2. pcc:ab,ti
3. cofact:ab,ti
4. beriplex:ab,ti
5. confidex:ab,ti
6. octaplex:ab,ti
7. kcentra:ab,ti
8. 'prothrombinex vf':ab,ti
9. prothrombinex:ab,ti
10. 'prothrombin complex':ab,ti
11. 'blood coagulation factors':ab,ti
12. blood:ab,ti AND coagulation:ab,ti AND factors:ab,ti
13. ppsb:ab,ti
14. 'ppsb sd':ab,ti
15. '4-factor':ab,ti OR 'four-factor':ab,ti OR '4f-pcc':ab,ti OR '4-pcc':ab,ti OR 'pcc4':ab,ti
16. '3-factor':ab,ti OR 'three-factor':ab,ti OR '3f-pcc':ab,ti OR '3-pcc':ab,ti OR 'pcc3':ab,ti
17. #19 OR #20 OR #21 OR #22 OR #23 OR #24 OR #25 OR #26 OR #27 OR #28 OR #29 OR #30 OR #31 OR #32 OR #33 OR #34

#Adults#

36. 'adult'/exp NOT 'child'/exp NOT 'adolescent'/exp NOT 'infant'/exp

#2010 – 2022#

[01-01-2010]/sd NOT [01-11-2022]/sd

#Total#

#18 AND #35 AND #36 AND [01-01-2010]/sd NOT [01-11-2022]/sd

**687 results**

### Search equation (clinicaltrials.gov)

1. Prothrombin complex concentrate OR Prothrombin concentrate complex

247 results

## Supplementary Material 3: Study PICO

### Patients

- Adult trauma patients with trauma-induced coagulopathy

### Interventions

- PCC as stand-alone treatment or with co-treatments

### Control

- Any intervention

### Outcomes

- Primary: in-hospital mortality
- Secondary: transfusion volume and deep venous thrombosis

## Supplementary Material 4: Forest plots for observational studies and RCT

### A: In-hospital mortality


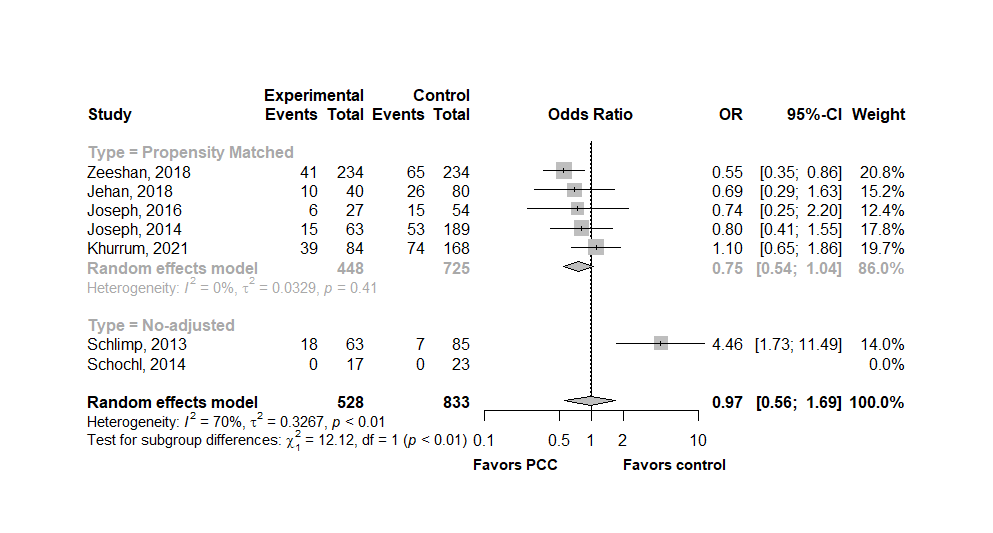


### B: Deep Venous Thrombosis


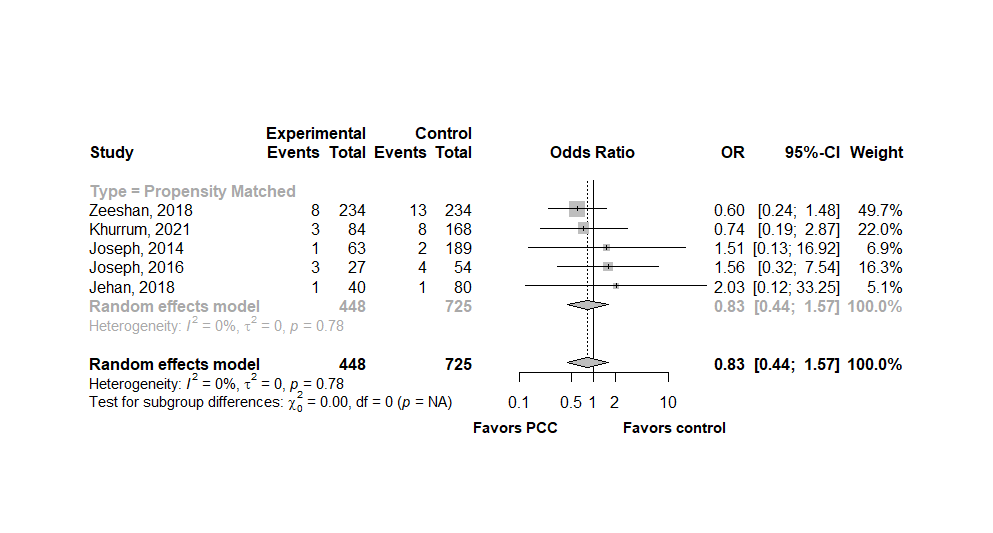


## Supplementary Material 5: Extraction table for the meta-analysis

In-hospital mortality

| **Studies** | **Adjusted** | **Control group** | | **Intervention group** | | **OR (95IC)** |
| --- | --- | --- | --- | --- | --- | --- |
|  |  | **Number of patients included in the analysis** | **Number of events** | **Number of patients included in the analysis** | **Number of events** |  |
| Joseph, 2014 | Yes, PS | 189 | 53 | 63 | 15 | 0.80 (0.41-1.55) |
| Joseph, 2016 | Yes, PS | 54 | 15 | 27 | 6 | 0.74 (0.25-2.20) |
| Zeeshan, 2018 | Yes, PS | 234 | 65 | 234 | 41 | 0.55 (0.36-0.86) |
| Jehan, 2018 | Yes, PS | 80 | 26 | 40 | 10 | 0.69 (0.29-1.63) |
| Khurrum, 2021 | Yes, PS | 168 | 74 | 84 | 39 | 1.10 (0.65-1.86) |
| Schlimp, 2013 | No | 85 | 7 | 63 | 18 | 4.46 (1.73-11.49) |
| Schöchl, 2014 | No | 23 | 0 | 17 | 0 | - |
| Bouzat, 2023 | RCT | 160 | 30 | 164 | 26 | 0.82 (0.46-1.45) |

Zeeshan (2019) was excluded of this analysis because it compared PCC in both groups (3F-PCC vs 4F-PCC). ORs: Odd Ratios; PS: Propensity Matched; RCT, Randomized Controlled trial

Deep Venous Thrombosis

| **Studies** | **Adjusted** | **Control group** | | **Intervention group** | | **OR (95IC)** |
| --- | --- | --- | --- | --- | --- | --- |
|  |  | **Number of patients included in the analysis** | **Number of events** | **Number of patients included in the analysis** | **Number of events** |  |
| Joseph, 2014 | Yes, PS | 189 | 2 | 63 | 1 | 1.51 (0.13-16.92) |
| Joseph, 2016 | Yes, PS | 54 | 4 | 27 | 3 | 1,56 (0.32-7.54) |
| Zeeshan, 2018 | Yes, PS | 234 | 13 | 234 | 8 | 0.60 (0.24-1.48) |
| Jehan, 2018 | Yes, PS | 80 | 1 | 40 | 1 | 2.03 (0.12-33.25) |
| Khurrum, 2021 | Yes, PS | 168 | 8 | 84 | 3 | 0.74 (0.19-2.87) |
| Bouzat, 2023 | RCT | 160 | 23 | 164 | 27 | 1.17 (0.64-2.15) |

ORs: Odd Ratios; PS: Propensity Matched; RCT, Randomized Controlled trial

## Supplementary Material 6: Risk of bias Traffic Light Plot

### *A*: Observational studies


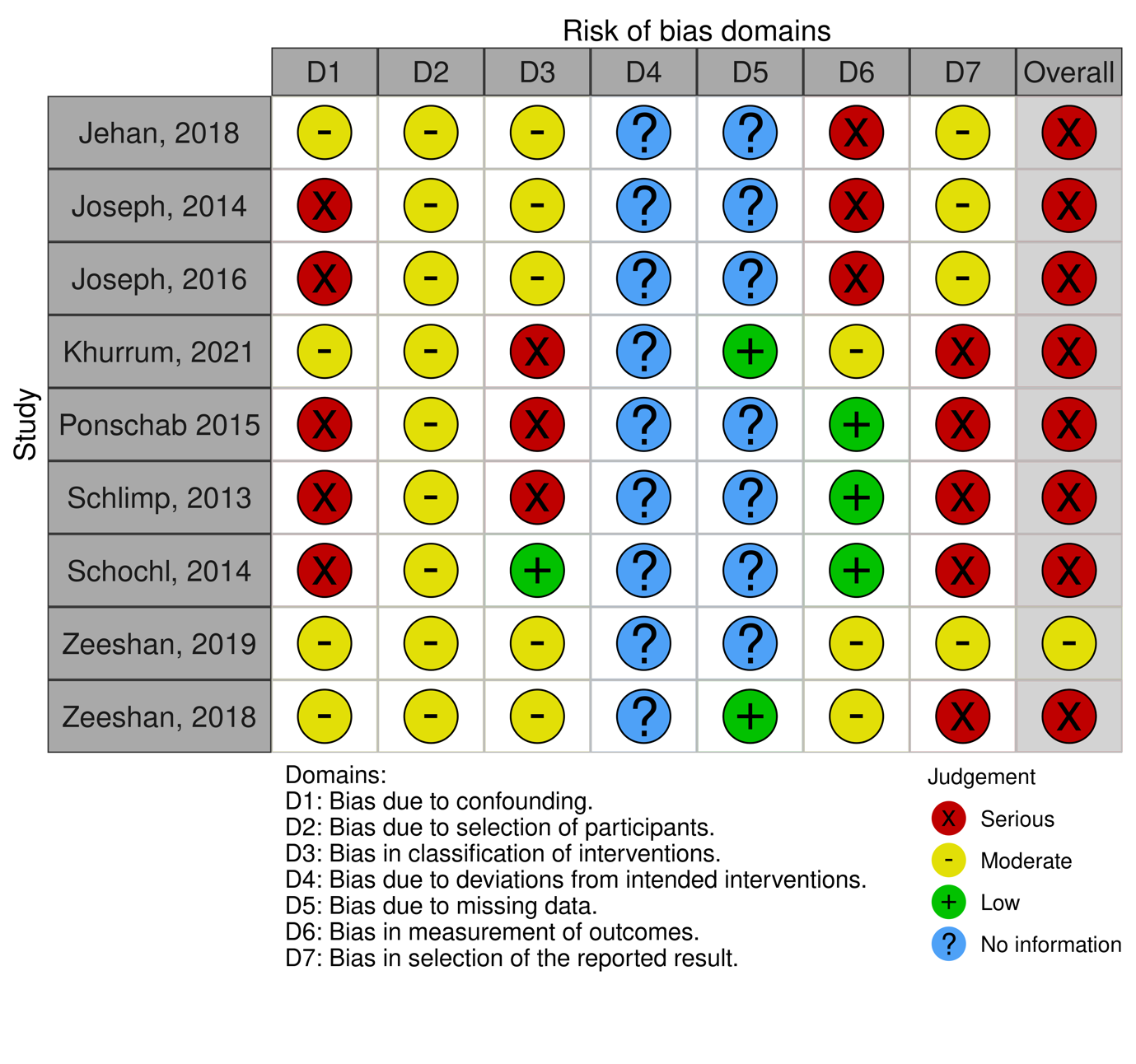


### B. Randomized controlled trial

###
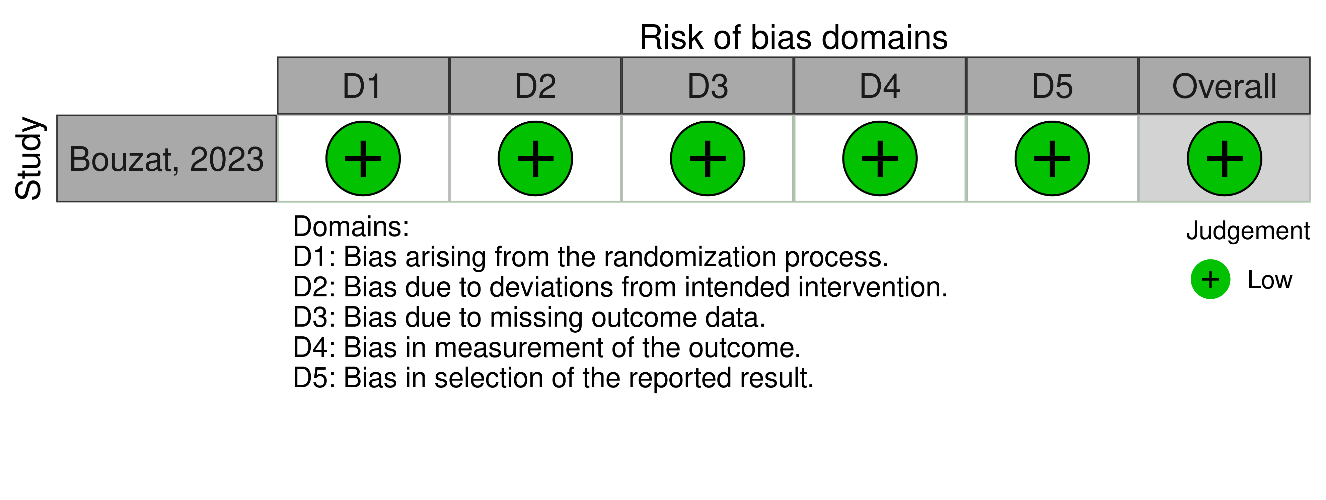


## Supplementary Material 7: Characteristics of included ongoing Randomized Controlled Trials

| **Identification** | **Title** | **Inclusion Criteria** | **Intervention** | **Control** | **Main Outcome** | **Status** |
| --- | --- | --- | --- | --- | --- | --- |
| NCT03981484 | REPlaCE (Randomized Trial Evaluating the Use of Prothrombin Complex concEntrate to Improve Survival in Patients with Traumatic Coagulopathy) trial | ≥15 years old, anticipate giving 4+ units based on Revised Assessment of Bleeding and transfusion score ≥ 2 | 4F PCC in addition to standard resuscitation methods | Site's standard resuscitation protocol: Whole Blood, Plasma, Packed Red Blood Cells | 24 hour mortality | Not yet recruiting |
| NCT04534751 | FiiRST 2 (Factors in the Initial Resuscitation of Severe Trauma 2) trial | Estimated age greater than 16 years old, severely injured (penetrating or blunt) trauma patients, triggered MHP within first hour of hospital arrival at the trauma bay/ED | FC (Fibryga) + 4F PCC (Octaplex) in the first and second massive haemorrhage protocol (MHP) packs. | Standard Frozen Plasma transfusion | Total ABP units (RBCs, FP, and platelets) transfused within 24 hours | Recruiting |
| NCT04019015 | Prehospital Kcentra for Hemorrhagic Shock | Trauma patients aged 18 years or greater, or weight > 50 kg if age unknown, systolic blood pressure < 70 mmHg, Suspicion of hemorrhagic shock based on mechanism of injury, EMS transport to a participating trauma centre | 4F PCC (Kcentra)  2000 U for patients with an estimated body weight ≤ 75kg  3000 U for patients with an estimated body weight > 75kg | A single infusion of volume matched placebo solution: NaCl 0.9% | Feasibility of PCC administration within 24 hours (Number of study drug kits opened and given to patients prior to hospital arrival) | Active, not recruiting |
| NCT05738642 | Evaluation of the Efficacy of Early Bunching of a FF-PCC in Patients With Severe Traumatic Hemorrhage | 18-80 yo and ISS≥16 and emergency previous class for 1 and 2 and within 3 hours after the trauma and Trauma induced active bleeding and ABC score ≥ 2 or blood loss ≥ 1000mL or ≥4 RBC expected to be transfused | Basic and normative treatment in accordance with the European Guidelines for Major Bleeding 2019: effective hemostatic measures and target-oriented correction of coagulation function. 2. 4F-PCC 25UI/Kg by intravenous infusion of cluster | Basic and normative treatment in accordance with the European Guidelines for Major Bleeding 2019: effective hemostatic measures and target-oriented correction of coagulation function. | Composite with the incidence of multiple organ failure within 7 days or mortality at 28 days | Not yet recruiting |
